# Uniparental disomy is a chromosomic disorder in the first place

**DOI:** 10.1186/s13039-022-00585-2

**Published:** 2022-02-17

**Authors:** Thomas Liehr

**Affiliations:** Institute of Human Genetics, Jena University Hospital, Friedrich Schiller University, Am Klinikum 1, 07747 Jena, Germany

**Keywords:** Uniparental disomy (UPD), Imprinting, Chromosome, Cytogenetics, Mosaic, Isodisomy, Heterodisomy

## Abstract

**Background:**

Uniparental disomy (UPD) is well-known to be closely intermingled with imprinting disorders. Besides, UPD can lead to a disease by ‘activation’ of a recessive gene mutation or due to incomplete (cryptic) trisomic rescue. Corresponding to all common theories how UPD forms, it takes place as a consequence of a “chromosomic problem”, like an aneuploidy or a chromosomal rearrangement. Nonetheless, UPD is rarely considered as a cytogenetic, but most often as a molecular genetic problem.

**Results:**

Here a review on the ~ 4900 published UPD-cases is provided, and even though being biased as discussed in the paper, the following insights have been given from that analysis: (1) the rate of maternal to paternal UPD is 2~3 to 1; (2) at most only ~ 0.03% of the available UPD cases are grasped scientifically, yet; (3) frequencies of single whole-chromosome UPDs are non-random, with UPD(16) and UPD(15) being most frequent in clinically healthy and diseased people, respectively; (4) there is a direct correlation of UPD frequency and known frequent first trimester trisomies, except for chromosomes 1, 5, 11 and 18 (which can be explained); (5) heterodisomy is under- and UPD-mosaicism is over-represented in recent reports; and (6) cytogenetics is not considered enough when a UPD is identified.

**Conclusions:**

As UPD is diagnosed using molecular genetic approaches, and thus by specialists considering chromosomes at best as a whim of nature, most UPD reports lack the chromosomal aspect. Here it is affirmed and substantiated by corresponding data that UPD is a chromosomic disorder in the first place and cytogenetic analyses is indicated in each diagnosed UPD-case.

**Supplementary Information:**

The online version contains supplementary material available at 10.1186/s13039-022-00585-2.

## Background

Nowadays, both DNA-based (= genetic) and epigenetic regulation are known to be essential for the correct function of a living cell. Thus, genetic and epigenetic alterations can lead to clinical problems in human, either via different or via interrelated metabolic pathways [1]. Genetic alterations may include DNA-sequence mutations and/or (sub-) chromosomal aberrations, detectable as deletions, duplications, insertions, inversions, and many other kinds of rearrangements [2]. Epigenetic changes typically showing up in connection with methylation defects on the DNA-double strand level [3], can also besides be due to altered chromosomal interphase-architecture, and/or appear secondary after an initial DNA-based mutation or (sub-) chromosomal aberrations [4, 5]. Interestingly, in 1993 Denise P. Barlow has proposed that genomic imprinting might have originally arisen from a host defense mechanism designed to inactivate retrotransposons [6].

Considering potentially disease-causing epigenetic alterations, primarily the phenomenon of (1) “genomic imprinting” is associated with and coming to each correspondingly educated person’s mind [1, 3]. However, it must be stressed, that also two other keywords are to be kept in mind besides, when considering epigenetic-related research and diagnostics: (2) uniparental disomy (UPD) and (3) cytogenetic alterations [7].

1. *Genomic imprinting* is happening, when one allele is silenced and only one stays active, and this monogenic expression in a diploid genome is strictly related to the parental origin. Imprinting is implemented by an epigenetic process, most often initiated by methylation of cytosines in a certain DNA-stretch. In case of exclusive presence of paternal or maternal imprinted allele(s) a corresponding syndrome may appear [1, 3]. The by now identified inherited genomic imprinting related disorders are listed in Table 1, as based on the literature [8–10]. Besides, genomic imprinting has been shown to play a role in tumorigenesis, too [11, 12].
2. *Uniparental disomy (UPD)* is the abnormal presence of either two paternal or two maternal homologous chromosomes in a disomic cell line. When such an event took place in an imprinted chromosome (Table 1), UPD is then the cause of the corresponding imprinting disorder/ syndrome [13].
3. *Cytogenetic alterations* and their impact on UPD-evolution are an underestimated and yet not really considered phenomenon in routine [7]. For Angelman syndrome one can find the statement that “fewer than 1% of individuals with Angelman syndrome have a cytogenetically visible chromosome rearrangement (e.g., translocation or inversion)” [14]. However, this reference does not provide any literature to underpin this. On the other hand, own studies [7] suggested, based on a literature review of then available 1,100 cases that at least 30% of constitutional UPD-events evolved due to a chromosomal aberration.

**Table 1:** The by now known imprinting disorders are detailed acc. to the literature [8–10]

Imprinting disorder	Gene(s) involved	Locus	Mosaic	Mechanisms				
				UPD	Dup	Del	Imprinting center defect	Single nucleotide variant
Transient neonatal diabetes mellitus (TNDM) (familial)	*PLAGL*:alt-TSS-DMR, LOM* *ZFP57*^+^	6q24	–	pat	6q pat	–	+	+
Birk-Barel intellectual disability syndrome (BBIDS)	*KCNK9*^+^	8q24	–	–	–	–	–	+
Silver-Russell syndrome (SRS)	*GRB10*:alt-TSS-DMR, GOM*	7	–	mat	7p and/or 7q mat	–	+	–
	*HG19/IGF2:*TSS-DMR, LOM* *KCNQ10T1:*TSS-DMR, GOM* *CDKN1C*^+^ *IGF2*^+^ *HMGA2*^+^ *PLAG1*^+^	11p15.5	+	mat	11p mat	–	+	+
Beckwith-Wiedemann syndrome (BWS)	*GRB10:*alt-TSS-DMR, GOM* *HG19/IGF2:TSS*-DMR, GOM* *KCNQ10T1:*TSS-DMR, LOM* *CDKN1C*^+^ mat	11p15.5	+	pat	11p pat	–	+	+
No syndrome yet but imprinting has been proven	*RB1*	13q14.2	–	mat	–	–	–	–
Temple syndrome (TS14)	*MEG3*/*DLK1*:TSS-DMR, LOM*	14q32	–	Mat	–	14q pat	–	+
Kagami-Ogata syndrome (KOS14)	*MEG3*/*DLK1*:TSS-DMR, GOM*	14q32	+	pat	–	14q mat	–	+
(familial) Central Precocious Puberty (CPPB)	*DLK1*^+^ mat	14q32	–	–	+	+	–	+
Prader-Willi syndrome (PWS)	*SNURF:*TSS-DMR, GOM*	15q11q13	+	mat	–	15q pat	–	+
Angelman syndrome (AS)	*SNURF:*TSS-DMR, LOM* *UBE3A*^+^ mat	15q11q13	+	pat	–	15q mat	+	+
Central Precocious Puberty 2 (CPPB2)	*MKRN3*^+^ pat	15q11.2	–	–	–	–	–	+
Schaaf-Yang syndrome (SHFYNG)	*MAGEL2*^+^ pat	15q11.2	–	–	–	–	–	+
Pseudo-hypoparathyoridism type 1B (PHP1B)	*GNAS-NESP:*TSS-DMR, GOM* *GNAS-AS1:*TSS-DMR, LOM* *GNAS-XL*:Ex1-DMR, GOM* *GNAS*^+^	20q13	+	pat	–	20q mat	+	+
Mulchandani-Bhoi-Conlin syndrome (MBCS)	n.a	6q24	–	mat	–	–	–	–
Multilocus imprinting disturbance (MLID)	They can show mixture of all above mentioned imprinting disorders or main features of only one of them							

Here, we did a reanalyzes of the yet available data—i.e. in almost 4900 UPD cases—to establish a more realistic view on the role of chromosomal rearrangements in early embryogenesis on UPD-formation and imprinting.

### Uniparental disomy (UPD): some basics

UPD was suggested to exist by Prof. Eric Engel in 1980, 7 years before the first molecular proven UPD case was published [15, 16]. In clinical cases, inborn UPD was yet reported for all 48 theoretically possible chromosomal (including 2 whole genomic variants), apart from maternal UPD of the Y-chromosome which biologically cannot exist—still, all 47,XYY carriers (and similar Y-chromosome doubling) have UPD(Y)pat [7, 17, 18]. As summarized in Table 2 normal/healthy carriers of single, whole-chromosome UPDs has been reported by now for all chromosomes (certainly without imprinting disease related ones—Table 1), apart from UPD(19)mat.

**Table 2:** All single-whole chromosome UPDs for which healthy carriers are reported; data acc. to Refs. [17, 18]

Chromosome #	Uniparental disomy in healthy carrier reported					
	Maternal		Paternal		Unclear	
	[18]	[17]	[18]	[17]	[18]	[17]
**1**	+	+	+	+	n.a	n.a
**2**	+	+	+	+	+	n.a
**3**	n.a	+	+	+	n.a	n.a
**4**	+	+	n.a	+	+	n.a
**5**	n.a	+	n.a	+	n.a	n.a
**6 (imprint. disord.)**	( +)	+	–	+	–	n.a
**7 (imprint. disord.)**	–	+	–	+	–	n.a
**8**	+	+	n.a	+	n.a	n.a
**9**	+	+	+	+	n.a	n.a
**10**	+	+	n.a	+	n.a	n.a
**11 (imprint. disord.)**	–	–	–	[ +]	–	n.a
**12**	( +)	+	n.a	+	+	n.a
**13**	+	+	+	+	+	n.a
**14 (imprint. disord.)**	–	[ +]	–	[ +]	–	n.a
**15 (imprint. disord.)**	–	[ +]	–	[ +]	–	n.a
**16**	+	+	(+)	+	+	n.a
**17**	+	+	(+)	+	n.a	n.a
**18**	(+)	n.a	(+)	n.a	n.a	n.a
**19**	n.a	n.a	n.a	+	+	n.a
**20 (imprint. disord.)**	(+)	[+]	–	n.a	–	n.a
**21**	+	+	+	+	+	n.a
**22**	+	+	+	+	n.a	n.a
**X**	+	+	+	+	+	n.a
**Y (XYY-syndrome)**	–*	n.a	(+*)	n.a	–*	n.a

– = no normal individuals available due to imprinting disorder; + = normal individuals reported; (+) = reported only in newborn; (+*) = reported only in male with two Y-chromosomes; –* = no reports as biologically not possible; [+] = should not exist—no data interpretation; imprint. disord. = imprinting disorder; n.a. = no reports available

UPD can be grouped into cases with pure isodisomy, pure heterodisomy and such with mixed iso-/heterodisomy. Also, one must distinguish UPD of a whole haploid chromosome set, UPD of one whole chromosome, and UPD of a part of a chromosome—a segmental UPD; the latter subtype is normally explained to be the consequence of a chromosomal rearrangement rescue-event [7].

Consequences of UPD can be threefold [7]:

- in case of isodisomy or heterodisomy an affected region underlying imprinting will inevitably and necessarily lead to the corresponding disorder as listed in Table 1;
- in case of isodisomy an affected region carrying a mutation in a recessive gene is correspondingly present in homozygote state; thus, this in a parent in heterozygote state ‘silent mutation’ is activated and leads to the corresponding rare disease; this may happen on chromosomes underlying imprinting and such not carrying imprinted genes, including father–son transmission of X-linked disorders.
- Besides, (cryptic) (sub)chromosomal aberrations present as mosaics or in all body cells may lead to UPD (for more details see below).

As previously stated: “UPD can be detected based on cytogenetic data and chromosomal heteromorphisms or rearrangements, microsatellite analysis, methylation test or SNP-based array-comparative genomic hybridization. Also, molecular cytogenetics taking advantage of the so-called copy number variations within the human genome can be used to characterize UPD” [7]. It must be stressed, that in single nucleotide polymorphism (SNP) -array only **isodisomy** can be detected and is normally blind for **heterodisomy**. As suggested by an exome-based sequencing approach, pure isodisomy, pure heterodisomy, mixed iso-/heterodisomy and segmental UPD show up in a mathematical relationship of 35 to 13 to 41 to 11 [19].

### The web-page “cases with uniparental disomy” (UPD)

Since 2008 the author of this paper maintains the web-page “cases with uniparental disomy (UPD)” [18]. The page has two aims: (1) collect all available case-reports on UPD in clinical cases published in peer-reviewed journals as listed in PubMed (https://pubmed.ncbi.nlm.nih.gov) and Google Scholar (https://scholar.google.com/); i.e. UPD in tumors as well as acquired but non-cancer-related disorders with UPD are not included; (2) provide information for patients and clinicians. This page included by 2010 ~1100 cases and by now 4879 cases with UPD; this means that per year ~ 350 UPD cases are published. As according to Nakka et al. [17] the UPD incidence is 1 in 2 × 10^3^, there are at present (with 8 × 10^9^ humans on this planet) there are ~16 million people with UPD alive; this means that less than 0.03% of UPD cases have found their way into the scientific data. Nonetheless, this is the yet available data, and a meta-analysis was done here to see what we can learn therefrom.

## Results

**Fig. 1:**
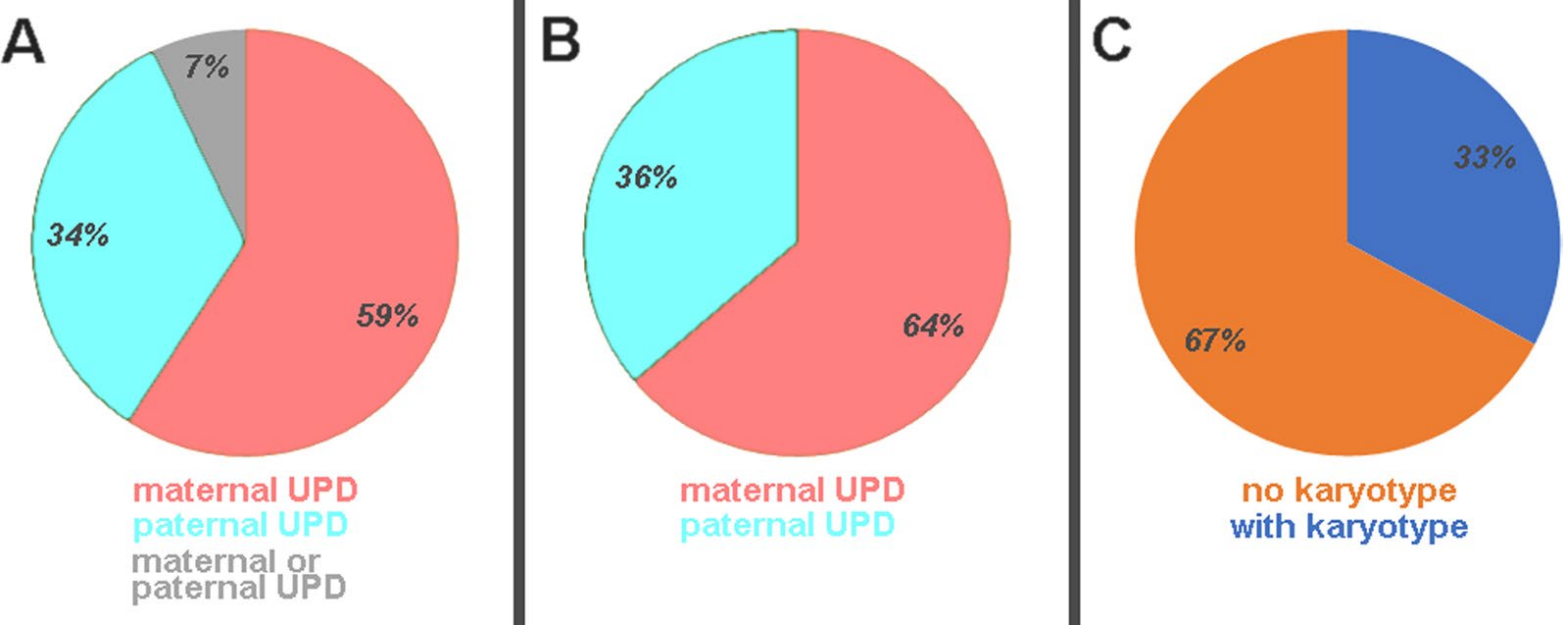
Distribution of 4879 cases with maternal and paternal UPD and UPD cases in which parental origin was not determined is shown in **A**. In **B** only those 4530 UPD cases are summarized for which parental origin was determined. In **C** for all 4879 it is shown when a karyotype was provided

The 4879 cases from Liehr [18] (Additional file 1) revealed a distribution of maternal versus paternal UPD cases (UPDmat: UPDpat) of roughly 2:1 (Fig. 1A, B). For 7% of the cases (349/4879) the parental origin was not reported (Fig. 1A); considering only the 4530 cases for which parental origin was available (2890 cases with UPDmat; 1640 with UPDpat) UPDmat was present in 67% and UPDpat in 33% (Fig. 1B). As shown in Fig. 1C only for 1605/4879 cases (33%) karyotypic information was available.

**Fig. 2:**
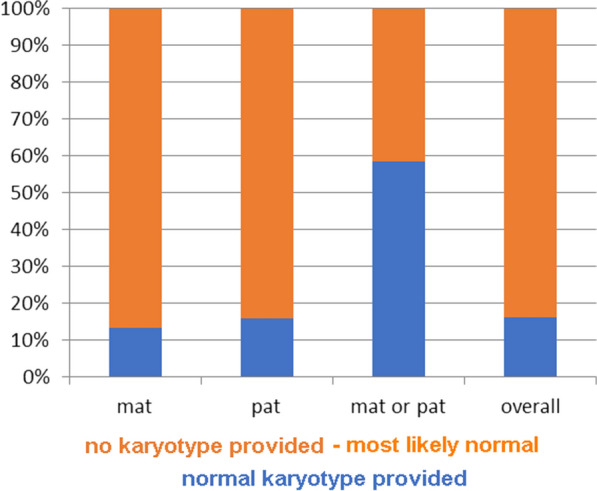
3901 UPD cases with presumably normal karyotype are shown, and in how many of the cases a normal karyotype was indeed reported as 46,XX or 46,XY. This data is broken down by parental origin of the UPD

**Fig. 3:**
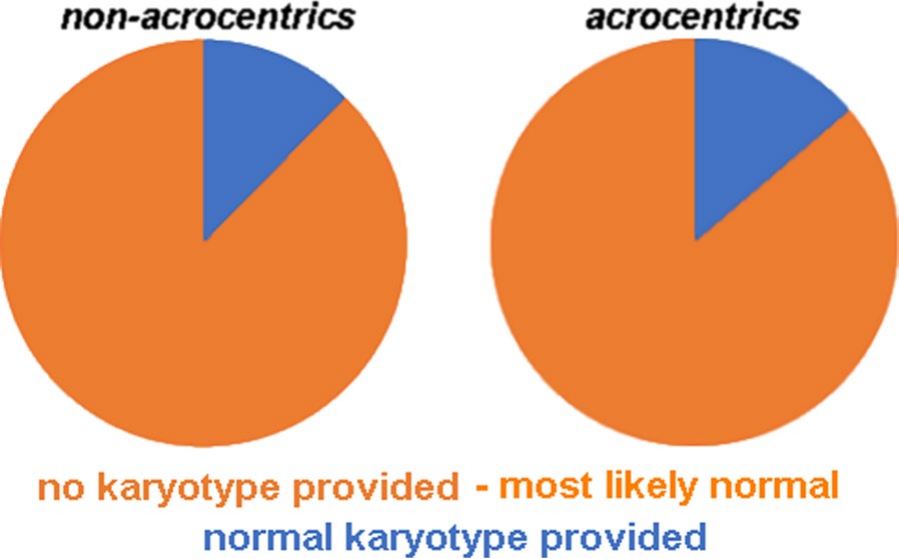
As before for Fig. 2, 3901 UPD cases with presumably normal karyotype are shown, and in how many of the cases a normal karyotype was indeed reported as 46,XX or 46,XY. This data is broken down for acrocentric and non-acrocentric chromosomal UPDs

Figure 2 shows in more detail the situation for the 3901 UPD cases with presumably normal karyotype. Only for 627/3901 cases (16%) a normal karyotype was reported. In the remainder, a karyotype was not even mentioned—i.e., it is not known if it was tested at all; however, according to the way the reports were presented, it had to be suggested that there was no obvious hint on a chromosomal imbalance to be considered. Interestingly, for those cases where parental origin of UPD could not be determined, it was more likely that the karyotype of the UPD patient was reported (Fig. 2). Furthermore, in case a normal karyotype was suggested, there was no difference in reporting a banding cytogenetic analysis if the UPD was associated with an acrocentric or a non-acrocentric chromosome (Fig. 3).

**Fig. 4:**
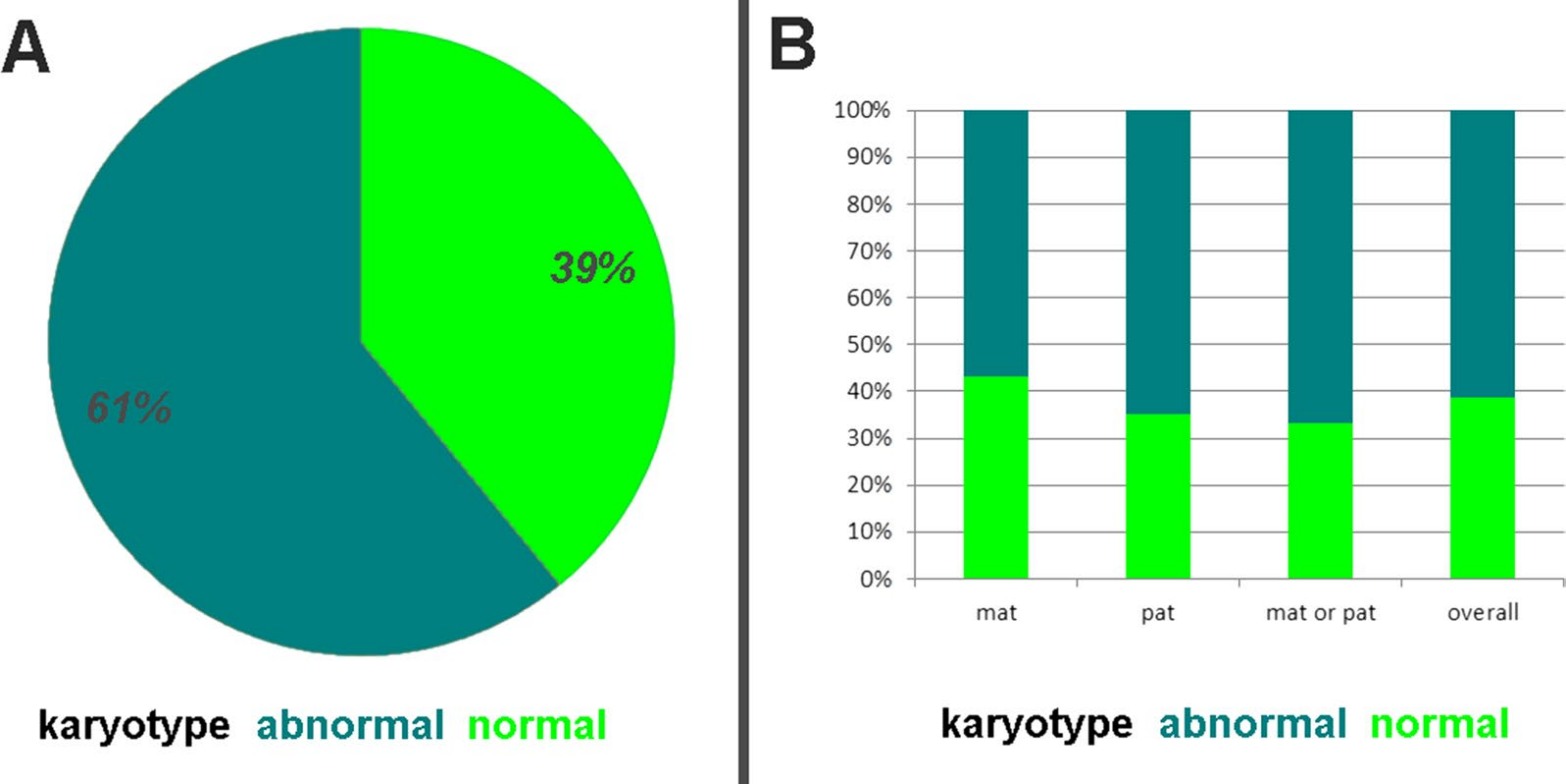
Only those 1605 UPD cases are included for which a karyotype was reported as normal or abnormal. In **A** data is summarized in a pie chart and in **B** it is shown also separated by parental origin of the UPD

In Fig. 4 only those 1,605 UPD cases are summarized for which a karyotype was done: here an ~ 3 to 2 ratio was found for cases with chromosomal aberrations versus such without (Fig. 4A). Figure 4B shows that there are no differences between the parental-origin-subgroups.

**Fig. 5:**
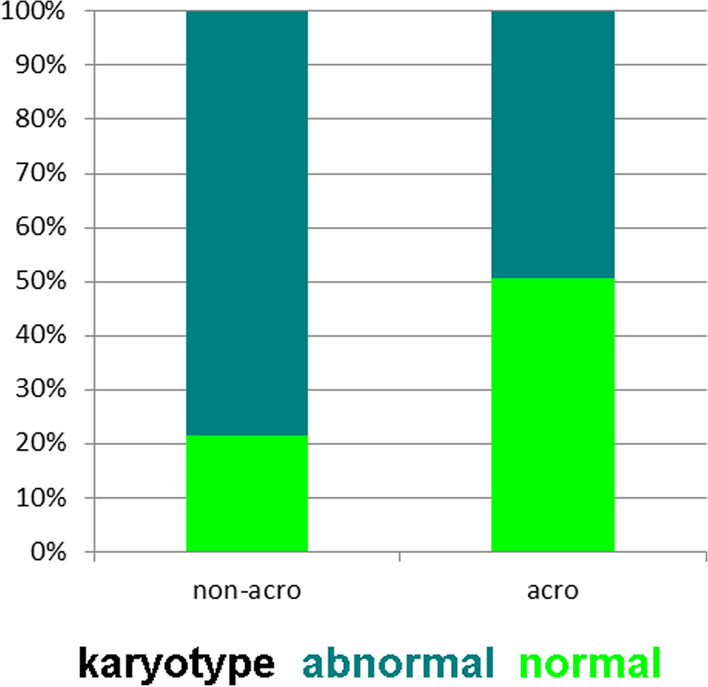
Karyotyping outcomes of acrocentric compared to non-acrocentric chromosomes based on 1455 cases are shown

In Fig. 5 the different karyotyping rates of acrocentric compared to non-acrocentric chromosomes are shown—here 1455 cases could be included. Abnormal karyotyping results were more likely to be found for UPD-cases connected with non-acrocentric (80%) compared to acrocentric chromosomes (50%).

**Fig. 6:**
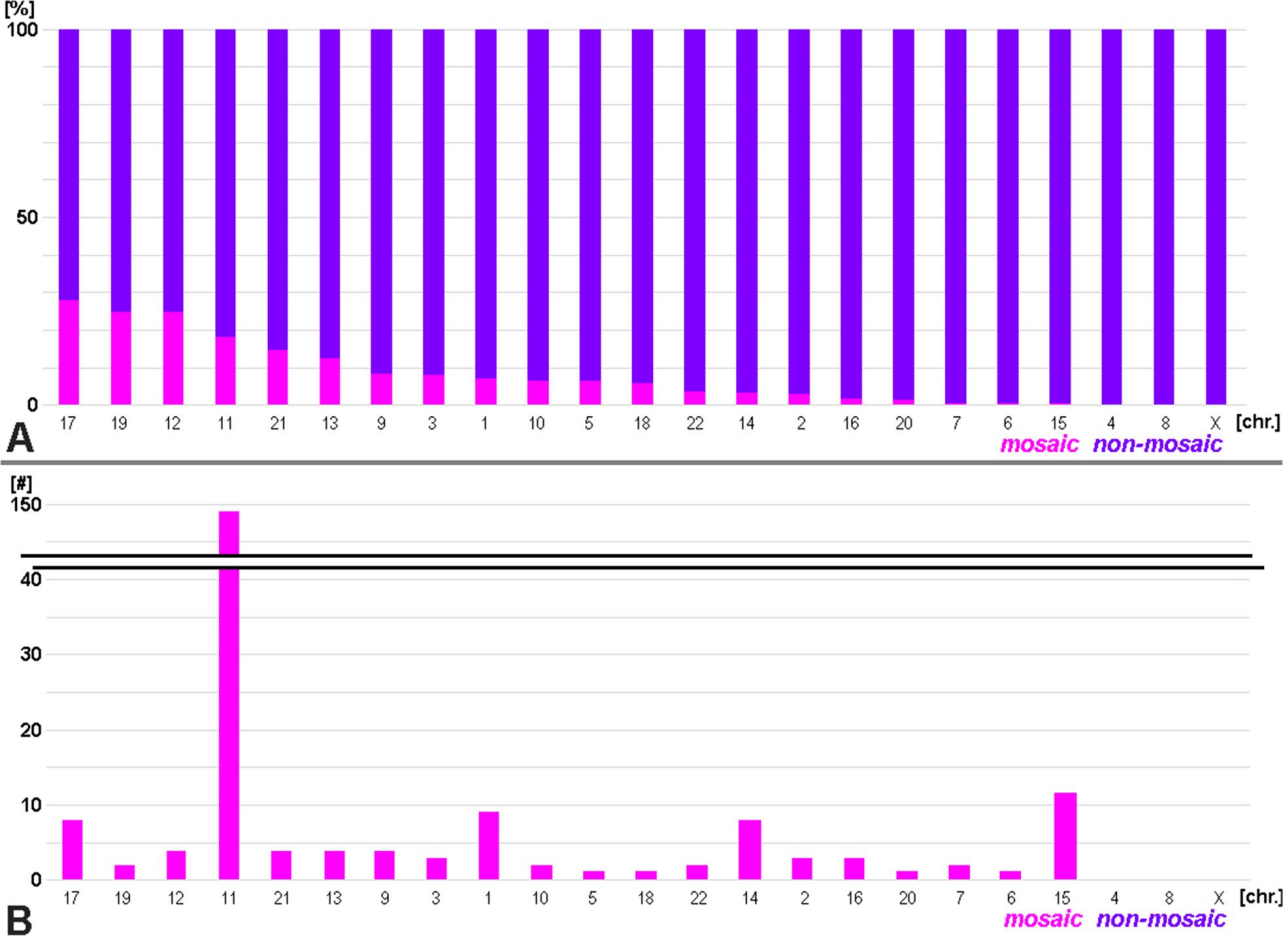
UPD can be present in all body cells or as a mosaic of affected and non-affected cells. In **A** cases with mosaic are shown by chromosome and sorted according to percentage of reported cases with UPD-mosaic; in **B** the same results are provided in absolute numbers of reported cases

The insight that UPD can be present in a carrier not in all body cells is a finding of the last decade, mainly [21]. 222/4541 (= 4.7%) of all yet reported cases are mosaic. The chromosome-specific distribution of reported UPD cases with and without mosaic are summarized in Fig. 6. Most frequently reported mosaic cases are among UPD of chromosomes 11, 15, 17, 19, 1 and 14—see also Additional file 2.

**Fig. 7:**
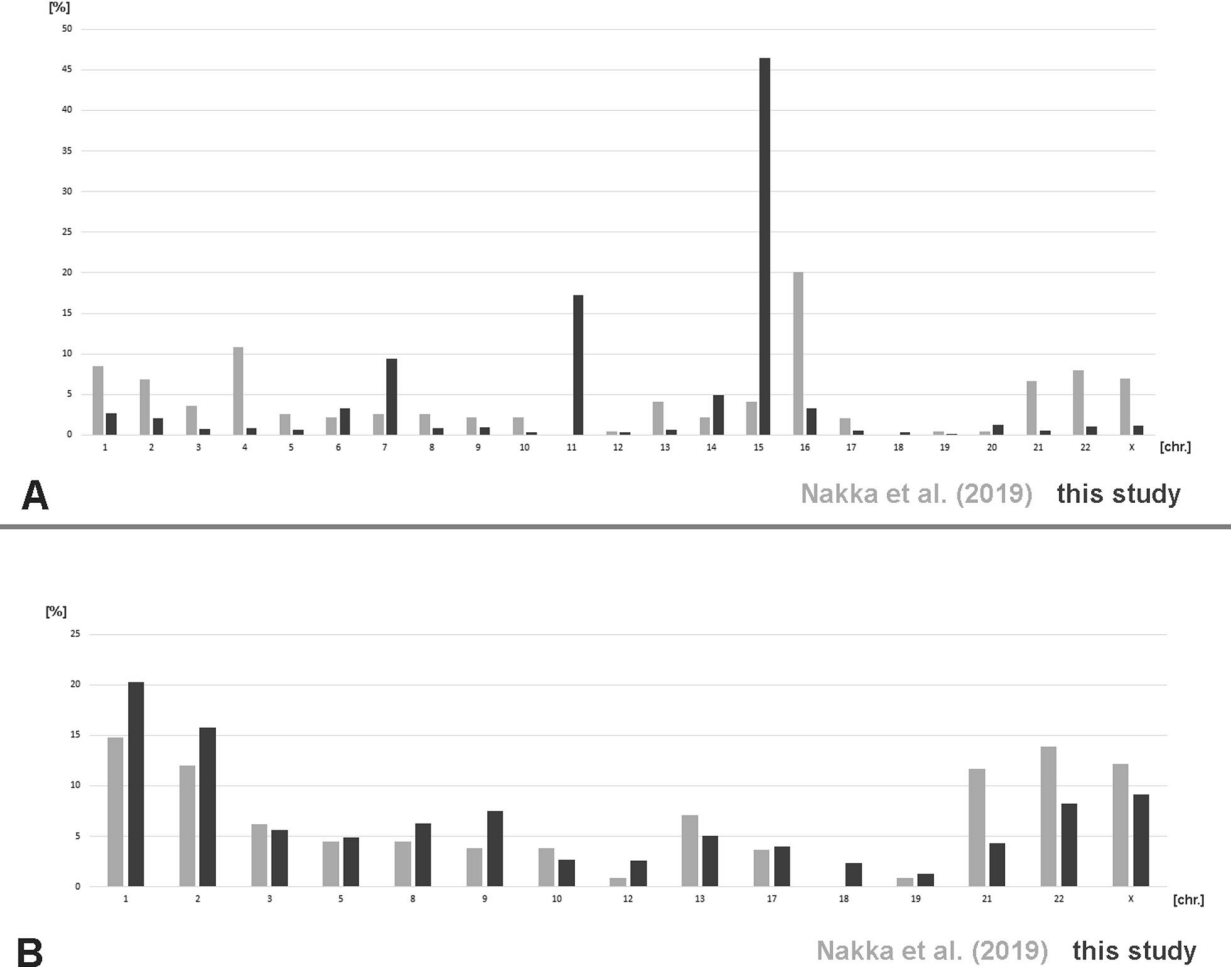
UPD frequencies by chromosomal origin according to this study [18] to a large single study [17]. In **A** results for all chromosomes are shown, in **B** only for those not being overrepresented in one of the two studies

Figure 7 summarizes the UPD frequencies by chromosomal origin from this database [18] and from the literature [17]. In Fig. 7A it is obvious that in the previously published study [17], UPD(4) and UPD(16) are overrepresented compared to the data from this study, while all imprinted chromosomes are underrepresented [18]. Comparing only frequencies for not imprinted chromosomes and also excluding UPD(4) and UPD(16) the detected UPD frequencies are within comparable ranges (Fig. 7B).

**Fig. 8:**
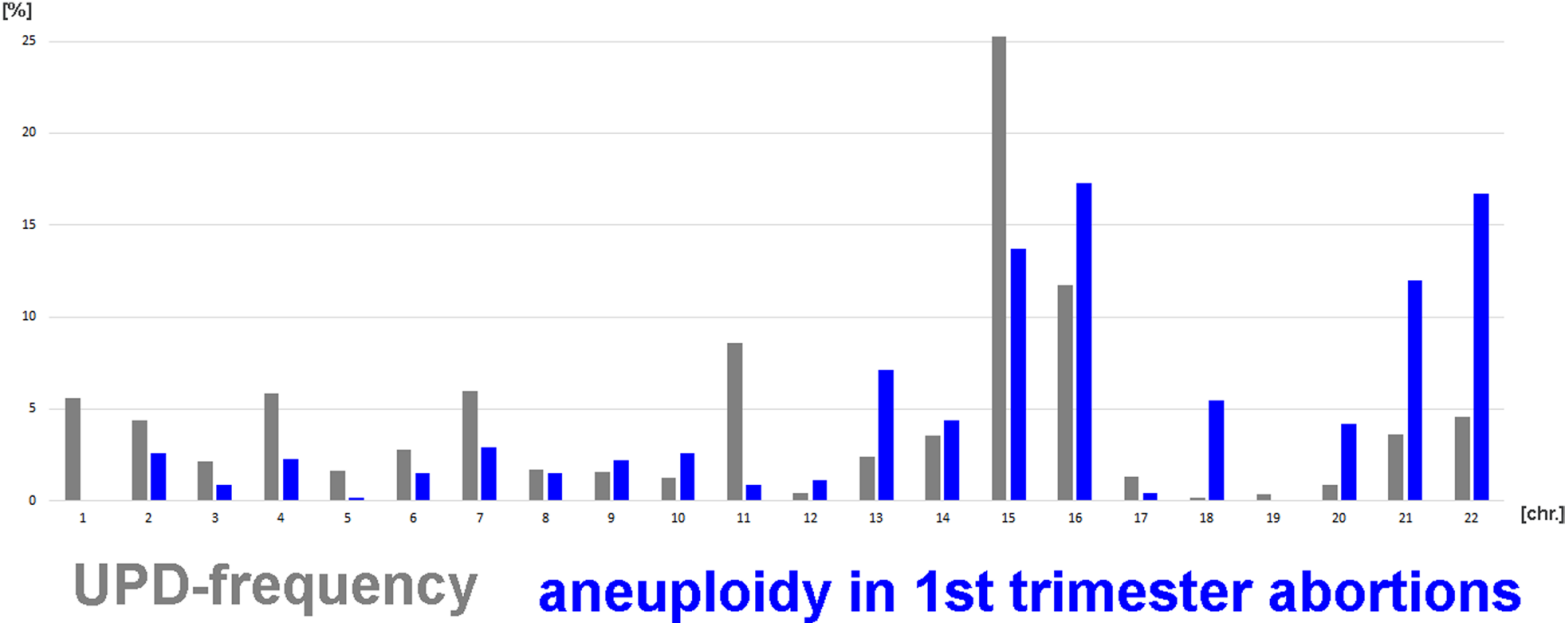
Overall frequencies for chromosomal UPDs from this [18] and a large single study [17] are compared to those for chromosomal distribution of aneuploidies in first trimester abortions [20]

When combining frequencies for chromosomal UPDs from the present study with that from the literature [17] and comparing those with the chromosomal distribution of aneuploidies in first trimester abortions [20], there is overall a striking overlap of the columns shown in Fig. 8; exceptions are chromosomes 1, 5 and 11 with less aneuploidies in first trimester abortions than UPD case and chromosome 18, vice versa.

Finally, for 34 of 306 Angelman syndrome patients with UPD (see Additional file 1), chromosomal rearrangements are reported—which are ~ 11% for all cases and 42% (34/81) including only those cases where a karyotype was reported explicit.

## Discussion

### What about the available dataset?

In the literature there are at present 4,879 constitutional UPD cases reported [18]. However, this data is biased in many ways.

There is (1) a *publishing bias*—i.e. mainly ‘interesting cases’ are published; this also includes the problem that as soon as more than ~50 case reports are available in literature it might be difficult to publish a single case and therefore cases might remain in the drawer;

(2) there is a *bias of ‘one hit wonder-reporting’*, i.e. UPD cases are published as single case studies of authors reporting the first UPD case they ever meet in their research carrier—thus important tests are not always done and/or information is missing to provide a comprehensive picture of the case;

(3) there is a *bias due to exclusively use of SNP-array to check for UPD*; it has been admitted that SNP-array testing misses at least 1/3 of UPD cases, as it only can detect isodisomy [22, 23]. This point is ignored in many SNP-array based UPD-reports, and as heterodisomy is not tested, reports of segmental UPD may be indeed reports of mixed iso-/heterodisomy and cases with heterodisomy are missed, as no analyses for both, iso- and heterodisomy, was done;

(4) there is the ‘*multiple publishing of a case without mentioning-bias’*; i.e. especially for cohort studies on UPD(7), UPD(11), UPD(14) and UPD(15) patients it can be suggested that the same, but also different authors may include partially or completely overlapping patients—however, the fact of patients being previously mentioned is not always given in those reports; and

(5) there is a *‘what is being tested-bias’*; i.e. UPD is mainly studied targeted in chromosomes underlying imprinting, as nicely highlighted by the complete different UPD-frequencies found in this review and a population based study for all imprinted and the non-imprinted chromosomes 4 and 16 (Fig. 7) [17].

Also, as outlined in Material and Method part, often the provided information about published UPD-cases is incomplete. Table 3 provides a checklist what would be ideally reported for each published inherited UPD case, to be really comprehensive.

**Table 3:** Checklist what optimally should be reported for each published UPD case

Checklist what needs to be provided when reporting a UPD case	
Include if there is/are	Mention also
A clinical phenotype	Gender and age of patient
Isodisomy, heterodisomy or mixed iso-/heterodisomy	Test performed and if there are restrictions in those
A segmental, whole chromosome or several chromosomes affecting UPD	
A chromosomal aberration detectable as underlying cause of the UPD event	
A mosaic present	Which tissue(s) was/were studied
Variant (mosaic) conditions in different tissues	
Previous study in which the patient was already mentioned/published	If this cannot be completely excluded

Ideally, when a UPD is reported it should be applied this checklist

### What are the insights from this study?

Considering the mentioned shortcuts, nonetheless the data reviewed from the “cases with uniparental disomy (UPD)” database [18] can provide the following insights and hints on discrepancies within the literature, which need to be clarified in future studies.

- Frequency of maternal and paternal derived UPD

As mentioned before, considering a UPD incidence of 1 in 2000 [17], this dataset includes less than 0.03% of available UPD cases (with ~ 16 million people with UPD in 8 × 10^6^ humans on this planet). According to this actual meta-analysis there is a 2 to 1 rate of UPD(mat) versus UPD(pat) for UPD cases being mainly found in clinically affected people, as previously suggested [7]. Nakka et al. [17] found for the general/ healthy population a 3 to 1 rate of UPD(mat) versus UPD(pat). Interestingly a meta-analysis of Eggenhuizen et al. [24] found a 9 to 1 rate of UPD(mat) versus UPD(pat) in clinical cases with confined placental mosaicism. This may be explained at first by the fact that trisomy is mostly due to nondisjunction in oocytes of elderly women. However, the zygote keeps the different parental genomes well separated [25], and thus in most cases can eliminate the additional chromosome from the correct parental genome. Thus, another possible explanation might be involving the idea of David Haig that “imprinted genes of maternal and paternal origin favor different degrees of proliferation of particular cell types in which they reside” [26]. According to him, paternal imprint favors placental growth (fathers are evolutionary interested in the actual pregnancy), and maternal imprint favors fetal growth (mother is interested in conserving her own resources to have this but also more future children). Applying this to the reported 9 to 1 rate of UPD(mat) versus UPD(pat) in clinical cases with confined placental mosaicism could mean that those pregnancies with UPD(pat) developed towards complete hydatidiform moles and ended in early abortions, while the UPD(mat) cases escaped from being partial hydatidiform moles, as maternal imprint in general provides support to both, placenta and fetus.

- Frequencies of single whole-chromosome UPDs are non-random
  Overall, UPD-formation is obviously a rare event: 1 in 2000 [17]. Nonetheless, according Fig. 7 UPD(4) and UPD(16) are more frequent in general population than suggested due to clinical studies, and imprinting related UPDs are overrepresented in the clinical case studies [18]. UPD(16) is the most frequent one in healthy humans and UPD(15) most likely the one with the highest incidence in syndromic patients. However, both studies are in concordance, that UPDs of chromosomes 1, 2, 3, 5, 8, 9, 10, 12, 13, 17, 21, 22 and X are relatively rare, and UPD(18) and UPD(19) are the most exotic UPD-conditions at all. The latter finding implies also that there is no correlation of UPD-formation with gene-density or positioning of a chromosome in the interphase nucleus, as chromosomes 18 and 19 are “the standard examples” for either a gene-poor chromosome located in the nuclear periphery or a gene-dense chromosome located in the center of the nucleus [5]. Also, chromosomal size cannot be involved in UPD-formation, as e.g. chromosomes 1, 16 and 21 constituting ~ 9%, 20% and 7% of UPD-cases [17] have chromosomal sizes of 250 to 90 to 48 Mb [27].
  A correlation with known frequent first trimester trisomies [20] and trisomic rescue leading to UPD of these chromosomes is obvious for most chromosomes (Fig. 8). It can be hypothesized that chromosome 1 and 5 trisomies have to be rescued at very early stages of pregnancy to be viable, and thus are rarely seen in (later) first trimester abortions [20]. Chromosome 11 UPDs have an exceptional high proportion of segmental UPDs [18], which might explain the discrepancy of the data for this chromosome. Why for chromosome 18, being one of the three most common viable human trisomies [2], hardly never UPD-cases are reported must be elucidated in future studies. Still, there are tumor related genes on this chromosome, especially tumorsuppressor genes, which could play a role in early embryogenesis the one or other way [28].
- Isodisomy, heterodisomy, mixed iso/heterodisomy, segmental UPD
  The different possible consequences of the isodisomy (including segmental UPD), heterodisomy and mixed iso/heterodisomy were treated already above. While before introduction of SNP based methods for UPD-analyses it was easy to extract from a report which kind of UPD was found, nowadays this becomes more and more difficult. One reason is lack of awareness for limitation to detect heterodisomy by SNP-based methods; another one is that in many benevolent screening studies in specific patient groups, testing of the parents is not included. If the latter would be done, shorter stretches of ‘loss of heterozygosity’ which just can be discussed as a potential UPD [29] could be solved in trio exome and/ or microsatellite-based approaches. More data on (mixed) iso- and heterodisomy could also provide to better understanding of chromosomal crossover events in meiosis [30].
- Somatic mosaicism in UPD – rule or exception?
  While heterodisomy is under-studied and under-reported nowadays, mosaicism in UPD can be picked up much better than ~ 10 years ago, both being due to increasing use of SNP-based UPD-detection [31]. Thus, mosaicism in UPD cases as summarized in Fig. 6 is for sure biased and under-detected, especially in older studies; it will turn out to be more common than just being present in about 5% of UPD cases. Thus, it is yet unclear if there is a chromosome-specific higher frequency for some selected chromosomes like chromosomes 11, 15, 17, 19, 1 and 14 (Fig. 6).
- What about cytogenetic changes in UPD-cases?

According to a previous review [7] and this study, only in 1/3 of the published UPD cases banding cytogenetics is done—or reported (Figs. 1C, 2). Even though a correlation of Robert’sonian translocations for acrocentrics and UPD is known [32] this rate does not differ between acrocentric and non-acrocentric chromosomes (Fig. 3). This is also evident as, even though, in contrast for what being stated for Angelman syndrome patients with UPD elsewhere [14] chromosomal rearrangements are identified in ~ 11 to 42% of the published cases acc. to this study. Furthermore, according to this meta-analysis ~ 60% of cytogenetically studied and published cases show chromosomal abnormalities, most of them being directly associated with the UPD (Figs. 4, 5).

Thus, looking at these data, as a (molecular) cytogeneticist, it seems to be clearly and openly visible to everyone that UPD is not a molecular genetic but a chromosomic disorder in the first place.

## Conclusion: there must be made a closer connection of UPD and chromosomal aberrations than presently done

Single whole-chromosomal UPD is thought to develop from trismic or monosomic rescue; segmental UPD is normally suggested to be due to a rescue-event of a balanced or unbalanced chromosomal rearrangement. Accordingly, UPD is in these modes of formation considered as a chromosome-based disorder. However, UPD is normally diagnosed by specialist being educated in molecular genetics, having in mind that genomic information is primarily encoded on DNA-level, where chromosomes do not play any role [33]. According to this meta-analysis at least 12%, maybe up to 61% of UPD cases have a detectable cytogenetic aberration, most of the reported ones being connected with the UPD event. This suggests a high necessity to do additional (cytogenetic) tests in case a UPD is identified. Most likely due to the mentioned focus on DNA level, 84% (3274–3901) of the UPD cases reported with presumably normal karyotype have either not been studied by cytogenetics or the cytogenetic studies were neither mentioned nor considered. Maybe the fact that mosaicism, being something typically being observed on chromosomal level, obviously plays a major role in UPD may help to understand that UPD is a chromosomic disorder of first cell divisions in the first place.

The latter was just recently highlighted by the finding that in ~ 30% of chromosomal UPD cases skewed X-chromosome inactivation was observed [34]. This means, that ~ 70% of rescue-events leading to a UPD happen before X-chromosome inactivation is initiated in 8 cell stage [35], and ~ 30% thereafter.

As shown in Fig. 8, there is a correlation of UPD and first trimester abortions, which also has already been suggested by scientific capacity in cytogenetics Albert Schinzel who stated that “the incidence of meiotic nondisjunction increases with advanced maternal age, maternal UPD most often is heterodisomy while in paternal UPD isodisomy prevails, and no correlation with paternal age is found” [36]. Accordingly, there is no doubt that UPD is a chromosomic disorder in the first place and UPD cases need always to be studied on chromosomal level to understand the biological processes ongoing in the individual patient the best.

## Material and methods

The 4879 constitutional UPD cases being bases for this study have been subdivided as follows in Liehr [18] and are correspondingly summarized in Additional file 1:

1. Cases with presumably normal karyotype, or nor karyotype reported, but a normal karyotype is obviously implied by the authors. These cases are subdivided in Additional file 1 in such a karyotype given and such without karyotype being explicit reported.
2. Cases with abnormal but balanced karyotype.
3. Cases with abnormal but unbalanced karyotype; segmental and whole chromosome UPD cases with balanced karyotype are included here, while cases with small supernumerary marker chromosomes (= sSMCs) are treated separately.
4. Cases with sSMC.
5. Cases with segmental UPD and no imbalances.

In the database itself [18] the cases are subdivided and specified beyond that in such UPD cases without, and such with clinical signs; as often clinical details lack within the latter group also such without clearly stated clinical correlation had to be included in the ones with clinical signs. Also, if a UPD is mosaic or present in all body cells of a carrier can be found on the subpages; data on non-mosaic-cases and mosaic cases was extracted in Additional file 2 concerning the chromosomal origin.

Data collected in Additional files 1 and 2 has been evaluated under different aspects as presented below in results-part. Besides, data for UPD frequencies of the database [18] was compared with the UPD frequencies found in 205 individuals in ~ 4 million people from normal population [17] and the distribution of chromosomal aneuploidies in first trimester abortions [20].

## Supplementary Information

**Additional file 1**. Detailed list of all in this study included UPD cases including chromosomal origin, parental origin of UPD, and chromosomal constitution.

**Additional file 2**. Detailed list of all in this study included UPD cases concerning mosaicism.

## Data Availability

The datasets generated and/or analyzed during the current study are available in the *Cases with uniparental disomy* repository (http://cs-tl.de/DB/CA/UPD/0-Start.html).

## Abbreviations

DNA: Deoxyribonucleic acid
mat: Maternal
Mb: Megabase
pat: Paternal
SNP: Single nucleotide polymorphism
UPD: Uniparental disomy
