# Supplementary material for: Uniparental disomy is a chromosomic disorder in the first place
Source: Mol Cytogenet. 2022 Feb 17;15:5. doi: 10.1186/s13039-022-00585-2 (PMC8851757; doi:10.1186/s13039-022-00585-2)
Supplement: Supplementary file 1 — Additional file 1. Detailed list of all in this study included UPD cases including chromosomal origin, parental origin of UPD, and chromosomal constitution. [file 13039_2022_585_MOESM1_ESM.docx]

|  | **no karyotype most likely normal** | | | **normal karyotype** | | | **abnormal  balanced  karyotype** | | | **abnormal  unbalanced karyotype** | | | **sSMC** | | | **segmental** | | | **all** | | | **IN SUMMARY** |
| --- | --- | --- | --- | --- | --- | --- | --- | --- | --- | --- | --- | --- | --- | --- | --- | --- | --- | --- | --- | --- | --- | --- |
| **chr.** | **mat** | **pat** | **uncl.** | **mat** | **pat** | **uncl.** | **mat** | **pat** | **uncl.** | **mat** | **pat** | **uncl.** | **mat** | **pat** | **uncl.** | **mat** | **pat** | **uncl.** | **mat** | **pat** | **uncl.** | **all** |
| **1** | *16* | *29* | *18* | *9* | *10* | *9* | *0* | *1* | *0* | *4* | *0* | *10* | *2* | *0* | *2* | *4* | *6* | *7* | **35** | **46** | **46** | **127** |
| **2** | *26* | *25* | *7* | *5* | *3* | *10* | *1* | *0* | *0* | *6* | *1* | *3* | *1* | *0* | *0* | *4* | *2* | *6* | **43** | **31** | **26** | **100** |
| **3** | *8* | *3* | *3* | *1* | *2* | *8* | *1* | *0* | *0* | *1* | *1* | *2* | *1* | *0* | *0* | *2* | *0* | *3* | **14** | **6** | **16** | **36** |
| **4** | *13* | *3* | *2* | *2* | *2* | *1* | *2* | *0* | *0* | *2* | *0* | *3* | *1* | *0* | *1* | *6* | *2* | *1* | **26** | **7** | **8** | **41** |
| **5** | *5* | *4* | *3* | *1* | *3* | *2* | *0* | *0* | *0* | *1* | *1* | *3* | *1* | *0* | *0* | *1* | *4* | *2* | **9** | **12** | **10** | **31** |
| **6** | *14* | *98* | *1* | *6* | *11* | *1* | *0* | *0* | *0* | *6* | *4* | *0* | *1* | *1* | *0* | *3* | *6* | *7* | **30** | **120** | **9** | **159** |
| **7** | *354* | *8* | *1* | *32* | *2* | *0* | *7* | *0* | *0* | *2* | *1* | *1* | *9* | *0* | *1* | *16* | *2* | *12* | **420** | **13** | **15** | **448** |
| **8** | *5* | *6* | *5* | *2* | *1* | *4* | *0* | *0* | *0* | *3* | *3* | *3* | *1* | *0* | *2* | *1* | *2* | *2* | **12** | **12** | **16** | **40** |
| **9** | *6* | *4* | *1* | *6* | *0* | *1* | *1* | *0* | *0* | *9* | *4* | *6* | *1* | *1* | *0* | *2* | *0* | *5* | **25** | **9** | **13** | **47** |
| **10** | *6* | *2* | *0* | *1* | *1* | *0* | *0* | *0* | *0* | *2* | *1* | *2* | *1* | *0* | *0* | *1* | *0* | *0* | **11** | **4** | **2** | **17** |
| **11** | *1* | *561* | *2* | *3* | *3* | *0* | *0* | *0* | *0* | *4* | *4* | *1* | *0* | *0* | *0* | *5* | *227* | *9* | **13** | **795** | **12** | **820** |
| **12** | *2* | *2* | *2* | *0* | *1* | *0* | *0* | *0* | *0* | *3* | *0* | *1* | *2* | *0* | *0* | *0* | *0* | *3* | **7** | **3** | **6** | **16** |
| **13** | *4* | *3* | *3* | *1* | *0* | *0* | *3* | *4* | *0* | *1* | *2* | *3* | *0* | *0* | *0* | *3* | *2* | *3* | **12** | **11** | **9** | **32** |
| **14** | *47* | *35* | *1* | *17* | *27* | *9* | *36* | *8* | *0* | *16* | *3* | *2* | *7* | *1* | *0* | *7* | *5* | *12* | **130** | **79** | **24** | **233** |
| **15** | *1558* | *225* | *2* | *222* | *47* | *18* | *25* | *21* | *0* | *30* | *3* | *9* | *27* | *7* | *0* | *3* | *3* | *11* | **1865** | **306** | **40** | **2211** |
| **16** | *35* | *9* | *6* | *8* | *2* | *15* | *0* | *0* | *0* | *62* | *3* | *8* | *2* | *0* | *0* | *1* | *0* | *6* | **108** | **14** | **35** | **157** |
| **17** | *4* | *1* | *0* | *1* | *0* | *0* | *2* | *0* | *0* | *1* | *0* | *3* | *0* | *0* | *0* | *4* | *2* | *7* | **12** | **3** | **10** | **25** |
| **18** | *0* | *2* | *2* | *0* | *1* | *1* | *0* | *0* | *0* | *2* | *1* | *1* | *1* | *0* | *0* | *1* | *0* | *3* | **4** | **4** | **7** | **15** |
| **19** | *0* | *1* | *2* | *0* | *1* | *1* | *0* | *0* | *0* | *0* | *0* | *1* | *0* | *0* | *0* | *0* | *0* | *2* | **0** | **2** | **6** | **8** |
| **20** | *19* | *12* | *4* | *2* | *1* | *4* | *0* | *0* | *0* | *3* | *1* | *0* | *4* | *1* | *0* | *1* | *9* | *1* | **29** | **24** | **9** | **62** |
| **21** | *3* | *1* | *1* | *5* | *2* | *0* | *2* | *2* | *0* | *6* | *2* | *2* | *0* | *0* | *0* | *0* | *0* | *1* | **16** | **7** | **4** | **27** |
| **22** | *10* | *2* | *2* | *0* | *2* | *10* | *5* | *2* | *0* | *5* | *1* | *4* | *2* | *1* | *1* | *1* | *0* | *4* | **23** | **8** | **21** | **52** |
| **X** | *2* | *2* | *0* | *3* | *5* | *1* | *1* | *0* | *0* | *29* | *9* | *3* | *0* | *0* | *0* | *2* | *1* | *0* | **37** | **17** | **4** | **58** |
| **Y** | *0* | *0* | *0* | *0* | *0* | *0* | *0* | *0* | *0* | *0* | *0* | *0* | *0* | *0* | *0* | *0* | *0* | *0* | **0** | **0** | **0** | **0** |
| **all chrs.** | *5* | *25* | *0* | *1* | *76* | *1* | *0* | *0* | *0* | *3* | *6* | *0* | *0* | *0* | *0* | *0* | *0* | *0* | **9** | **107** | **1** | **117** |
| **summary** | **2143** | **1063** | **68** | **328** | **203** | **96** | **86** | **38** | **0** | **201** | **51** | **71** | **64** | **12** | **7** | **68** | **273** | **107** | **2890** | **1640** | **349** | **4879** |
