# Supplementary material for: Uniparental disomy is a chromosomic disorder in the first place
Source: Mol Cytogenet. 2022 Feb 17;15:5. doi: 10.1186/s13039-022-00585-2 (PMC8851757; doi:10.1186/s13039-022-00585-2)
Supplement: Supplementary file 2 — Additional file 2. Detailed list of all in this study included UPD cases concerning mosaicism. [file 13039_2022_585_MOESM2_ESM.docx]

| **chr.** | **mosaic** | | | | **non-mosaic** | | | |
| --- | --- | --- | --- | --- | --- | --- | --- | --- |
|  | **mat** | **pat** | **uncl.** | *overall* | **mat** | **pat** | **uncl.** | *overall* |
| **1** | *2* | *0* | *7* | *9* | *33* | *46* | *39* | *118* |
| **2** | *0* | *0* | *3* | *3* | *43* | *31* | *23* | *97* |
| **3** | *1* | *0* | *2* | *3* | *13* | *6* | *15* | *34* |
| **4** | *0* | *0* | *0* | *0* | *26* | *7* | *8* | *41* |
| **5** | *0* | *0* | *2* | *2* | *9* | *12* | *8* | *29* |
| **6** | *1* | *0* | *0* | *1* | *29* | *120* | *9* | *158* |
| **7** | *0* | *0* | *2* | *2* | *420* | *13* | *13* | *446* |
| **8** | *0* | *0* | *0* | *0* | *12* | *12* | *16* | *40* |
| **9** | *0* | *1* | *3* | *4* | *25* | *8* | *10* | *43* |
| **10** | *0* | *1* | *0* | *1* | *11* | *3* | *2* | *16* |
| **11** | *6* | *137* | *6* | *149* | *7* | *658* | *6* | *671* |
| **12** | *1* | *0* | *3* | *4* | *6* | *3* | *3* | *12* |
| **13** | *0* | *0* | *4* | *4* | *12* | *11* | *5* | *28* |
| **14** | *1* | *3* | *4* | *8* | *129* | *76* | *20* | *225* |
| **15** | *6* | *4* | *2* | *12* | *1859* | *302* | *38* | *2199* |
| **16** | *0* | *0* | *3* | *3* | *108* | *14* | *32* | *154* |
| **17** | *1* | *1* | *5* | *7* | *11* | *2* | *5* | *18* |
| **18** | *0* | *0* | *1* | *1* | *4* | *4* | *6* | *14* |
| **19** | *0* | *0* | *2* | *2* | *0* | *2* | *4* | *6* |
| **20** | *0* | *0* | *1* | *1* | *29* | *24* | *8* | *61* |
| **21** | *1* | *0* | *3* | *4* | *15* | *7* | *1* | *23* |
| **22** | *0* | *0* | *2* | *2* | *23* | *8* | *19* | *50* |
| **X** | *0* | *0* | *0* | *0* | *37* | *17* | *4* | *58* |
| **Y** | *0* | *0* | *0* | *0* | *0* | *0* | *0* | *0* |
| **summary** | **20** | **147** | **55** | *222* | **2861** | **1386** | **294** | *4541* |
